# The Joint Effects of Spatial Cueing and Transcranial Direct Current Stimulation on Visual Acuity

**DOI:** 10.3389/fpsyg.2018.00159

**Published:** 2018-02-19

**Authors:** Taly Bonder, Daniel Gopher, Yaffa Yeshurun

**Affiliations:** ^1^Department of Industrial Engineering and Management, Technion – Israel Institute of Technology, Haifa, Israel; ^2^Department of Psychology, University of Haifa, Haifa, Israel

**Keywords:** tDCS, spatial attention, visual acuity, Landolt, visual cortex

## Abstract

The present study examined the mutual influence of cortical neuroenhancement and allocation of spatial attention on perception. Specifically, it explored the effects of transcranial Direct Current Stimulation (tDCS) on visual acuity measured with a Landolt gap task and attentional precues. The exogenous cues were used to draw attention either to the location of the target or away from it, generating significant performance benefits and costs. Anodal tDCS applied to posterior occipital area for 15 min improved performance during stimulation, reflecting heightened visual acuity. Reaction times were lower, and accuracy was higher in the tDCS group, compared to a sham control group. Additionally, in post-stimulation trials tDCS significantly interacted with the effect of precuing. Reaction times were lower in valid cued trials (benefit) and higher in invalid trials (cost) compared to neutrally cued trials, the effect which was pronounced stronger in tDCS group than in sham control group. The increase of cost and benefit effects in the tDCS group was of a similar magnitude, suggesting that anodal tDCS influenced the overall process of attention orienting. The observed interaction between the stimulation of the visual cortex and precueing indicates a magnification of attention modulation.

## Introduction

We grow and learn through interaction with the world we live in. Efficient perception of our surrounding lays a keystone for cognition, decision making, and proceeding actions. Making sense of a visual scene we detect and identify information which holds a potential to promote our thinking. To enable this challenging process, visual attention serves as a guide for exploration, stretching a thread between the objective input and the subjective goals. Engagement of visual attention was shown to improve performance in various visual processes such as crowding (e.g., Yeshurun and Rashal, 2010), texture segmentation (e.g., Yeshurun and Carrasco, 1998) acuity, and hyperacuity (e.g., Yeshurun and Carrasco, 1999).

For example, Yeshurun and Carrasco (1999) examined the mechanisms through which spatial covert (transient) attention improves visual acuity. Allocating attention by a peripheral precue they found a significant improvement in observers' performance, which was manifested in faster reaction times and increased accuracy. Eliminating alternative explanations (i.e., diminished spatial uncertainty and changes in decisional factors) the authors deduced that allocation of attention to the target location enhances visual acuity, improving the quality of sensory representation. Following this line of research, Montagna et al. (2009) studied the effects of covert attention on visual acuity. They used a Landolt gap resolution task, in which two squares, each with a small gap either on its top or bottom side, were presented for a short duration, and were followed by a response cue. Participants were required to detect the side of the gap in the square which was indicated by the response cue. Attention allocation was manipulated by pre-cuing the location of one of the squares in advance. In the valid condition the precue and response cue indicated the same square, whereas in the invalid condition the precue and response cue indicated different squares. They found acuity trade-off between valid and invalid conditions: attention increased visual acuity at valid locations and decreased it at invalid locations, relative to a neutral baseline. The authors suggested that these findings imply a presence of visual acuity trade-off: the more processing resources are allocated to the attended location, the less resources are allocated to the unattended location. Thus, limited processing resources affect early vision by enhancing or reducing sensory representation in a pre-cued location (Montagna et al., 2009).

Neurocognitive studies offer an approach by which modifying activation in a specific brain area enables to test the resultant changes in performance. Such neuromodulatory techniques afford new insights into visual processes (Antal et al., 2001). Among these techniques stands transcranial Direct Current Stimulation (tDCS). tDCS uses weak direct currents to induce cortical activity by promoting reduction (cathodal stimulation) or enhancement (anodal stimulation) of neural excitability. tDCS differs qualitatively from other brain stimulation techniques, such as Transcranial magnetic stimulation—TMS, as it does not produce neural action potentials. Instead, the weak direct currents alter the resting state of neurons membrane potential, influencing the probability of spontaneously generated neural firing (Creutzfeldt et al., 1962). tDCS was shown to reliably modify cortical functioning, inducing focal, prolonged yet reversible shifts of cerebral excitability. Thus, the technique provides invaluable insights on the correlation between modification of behavior and its underlying neurophysiologic foundations. In addition, it is suggested that coupling tDCS with a behavioral task increases the specificity of stimulation, which might produce long-lasting effects (reviewed by Costa et al., 2015).

tDCS effects are studied in a wide range of research areas including motor rehabilitation, clinical psychology, cognition, and perception. In the field of visual perception, tDCS was shown to improve numerous visual abilities such as contrast sensitivity, color discrimination, spatial frequency identification, perceptual learning, and visual memory consolidation (reviewed by Nitsche et al., 2008). For instance, inspecting the influence of tDCS on the posterior occipital area, Sczesny-Kaiser et al. (2016) found an improvement in learning in a phosphene thresholds task, Costa et al. (2012) demonstrated enhanced chromatic contrast sensitivity, and Olma et al. (2011) revealed an improvement in orientation sensitivity. Moreover, a recent study performed by Reinhart et al. (2017) showed a significant improvement in visual hyperacuity, measured with Vernier stimuli. Relying on these demonstrations of improved neural processing as a result of anodal stimulation to posterior occiput, the current study examined whether this technique would also improve visual acuity measured with a Landolt gap task.

In addition, according to Guo et al. (2007) a change of attentional state affects the activity of sensory neurons in visual cortex. Consistently, there is evidence that feedback originating in higher-level areas can modify responses of primary visual cortex, accounting for attentional effects (e.g., Kastner et al., 1999). This raises the question whether an enhancement of early visual cortex processing can influence attention allocation. Indeed, Mulckhuyse et al. (2011) used a spatial cueing orientation discrimination task and showed that certain TMS intensities can amplify cueing effects, increasing facilitation for valid cues and interference for invalid cues, in a contralateral compared to ipsilateral visual field, relative to the stimulated visual area. Unlike TMS, anodal tDCS applied to early visual cortex does not induce action potentials but merely intensifies the organic brain functioning, highlighting the processes of interest. As to our knowledge the combined effects of attention and tDCS on visual acuity were not studied before, the aim of this study was to examine the mutual influences of attentional facilitation and concurrent tDCS on visual acuity. Specifically, we examined whether anodal tDCS applied to posterior occipital area will interact with the allocation of attention in a visual acuity task.

### Experimental paradigm and hypotheses

Landolt gap discrimination task combined with attentional precues was employed to study the joint effects of exogenous spatial attention and neurostimulation. Following Montagna et al. (2009), we included in our design valid, neutral and invalid cueing conditions. This enabled us to inspect both attentional costs and benefits. We chose a Landolt gap task because it is considered to involve early visual areas. Contrary to more complicated visual tasks, in which significant improvement is produced after extensive perceptual learning, in this basic task learning asymptote is achieved quickly, and typically no improvement is found after the initial practice (Westheimer, 2001). According to Fiorentini and Berardi (1981), objective 2-Alternative-Forced-Choice tasks, such as the Landolt gap task, show a learning effect that develops over ~200 trials. Hence, applying neurostimulation during this period of time enabled us to modulate the crucial period of participants' initial encounter with the task.

Relying on findings of Yeshurun and Carrasco (1999) and Montagna et al. (2009), we expected to reproduce the attentional effects on visual acuity. Additionally, given previous tDCS findings (Antal et al., 2003; Olma et al., 2011; Costa et al., 2012; Sczesny-Kaiser et al., 2016; and Reinhart et al., 2017) we hypothesized that anodal tDCS to the occipital area will improve visual processing during stimulation. Finally, combining attentional allocation with tDCS we postulated that the anodal stimulation may modulate the effects of cueing, influencing the effectiveness of spatial attention. We manipulated exogenous attention—an involuntary and automatic process, which is known to affect early stages of visual processing (e.g., Yeshurun and Carrasco, 1999). Two experiments were performed in order to test these hypotheses. Experiment 1 was designed to replicate the effects of spatial cueing on visual acuity, while Experiment 2 tested the joint effects of tDCS and attention allocation.

## Experiment 1

### Method

#### Participants

Fourteen undergraduates (mean age = 24.6, 9 female) from the University of Haifa participated in the experiment, all with normal or corrected to normal vision, right handed and naive to the purpose of the study. All the participants signed a written informed consent according to the Declaration of Helsinki guidelines, as approved by the institutional ethics committee of the University of Haifa (307/15).

#### Apparatus

The stimuli were presented using E-prime™ on Windows powered computer. The stimuli appeared on a 21 inch CRT color monitor set to resolution of 1,024 × 768 pixels with a refresh rate of 60 Hz. The participants sat 57 cm away from the computer monitor and viewed the display binocularly. They responded by pressing a key on a computer keyboard with the index or the middle finger of their right hand.

#### Stimuli and procedure

**Figure 1:**
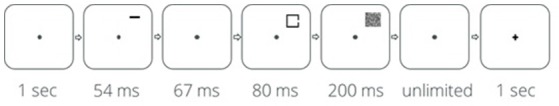
Single trial design.

The target was a black square presented on a white background for 80 ms. This stimulus duration kept the overall performance level at 75–85% correct, so that ceiling or floor effects would be avoided, and eye movements would be precluded (Mayfrank et al., 1987). The square appeared in one of two possible locations—the upper or the lower right side of the visual field, at eccentricity of 6° from the center of the display (Figure 1). The square subtended 1 × 1° of visual angle and contained a gap of 0.1° in one of its sides—left or right, with equal probability. The participants were asked to indicate, as rapidly and accurately as possible, which side of the square contained the gap. A 1.4 × 1.4° square of distorted lines served as the mask and was presented after the square's disappearance, at its location, for 200 ms. Following the mask, at the end of each trial, a plus (0.33° height 0.33° width) or a minus (0.33° width 0.14° height) black sign served as feedback, and was presented in the center of the display for 1,000 ms. A black fixation dot (0.15° diameter) was presented in the center of the screen throughout the trial, and the participants were asked to fixate it.

Prior to the square's appearance, a precue appeared for 54 ms, and after an Inter-Stimulus-Interval (ISI) of 67 ms the square was presented. One third of the trials were valid trials—the precue indicated the location in which the square was about to appear. To prevent masking effects, the precue appeared 0.3° above the location of the target. The precue was a green (0, 128, 0 in standard RGB color space) horizontal bar, subtending 0.5 × 0.14° of visual angle. Another third of the trials were invalid trials—the horizontal bar appeared above the other location (i.e., the location in which the square did not appear). The rest of the trials included a neutral cue—two identical bars were presented simultaneously, each above one of the possible target locations. The bars indicated that the square may appear at each location with equal probability. Square location, gap side, and precue type were randomized across trials.

The experimental session contained practice and test phases. The practice phase included 10 blocks of 20 trials (200 trials overall) and was followed by a test phase with 25 blocks of 20 trials (500 trials overall), for a total of 700 trials. Each block was followed by a feedback report of accuracy ratio achieved in it, and then by a short break which was terminated by the participant. Overall, the experimental session lasted for about 1 h.

#### Results

**Figure 2:**
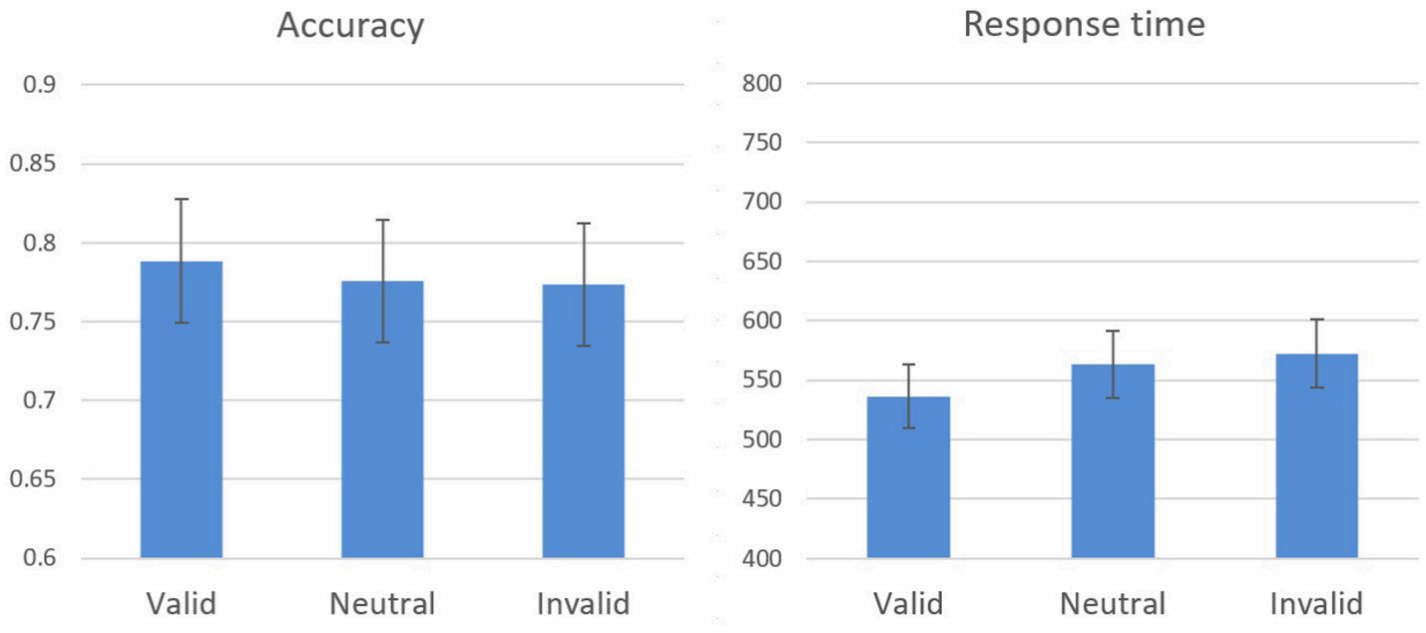
Experiment 1 results: response time and accuracy means and standard errors in the cueing conditions during the test stage.

The effects of spatial cueing on response time and on accuracy during the test phase were analyzed. Response time for correct answers only was taken into account, and outliers were omitted beyond 2 SD for each participant in each block. A within-subjects one-way analysis of variance (cueing condition: valid, neutral, invalid) performed on response time revealed a significant difference between the cueing conditions [*F*_(2, 26)_ = 7.85, *p* = 0.002, η^2^ = 0.38; Figure 2]. Attentional benefit was expressed in better performance [*t*_(13)_ = 3.02, *p* = 0.01] in valid trials [*M* = 536.49, *SE* = 4.79) compared to neutral trials (*M* = 563.08, *SE* = 5.32), while attentional cost, the difference between invalid (*M* = 572.27, *SE* = 4.88) and neutral trials, was found to be insignificant [*t*_(13)_ = 1.70, *p* = 0.11].

A similar analysis performed on accuracy did not reveal a significant effect [*F*_(2, 26)_ = 0.87, *p* = 0.43], however, accuracy changed as a function of cueing condition in the expected direction indicating that there were no speed-accuracy tradeoffs. Hence, these results resemble previous findings (e.g., Yeshurun and Carrasco, 1999), demonstrating the influence of spatial attention on visual acuity.

## Experiment 2

The goal of this experiment was to examine the combined influence of tDCS to the occipital cortex and attentional cueing on spatial resolution, measured by Landolt gap task. Two groups participated in this experiment. The tDCS group received anodal stimulation, while the control (sham) group received sham tDCS. Given the studies described in the introduction, we expected to find main effects for both the manipulation of spatial attention and tDCS stimulation. The critical question was whether an interaction will be found between these two factors.

### Method

#### Participants

Thirty undergraduates from The University of Haifa (Mean age = 26.3, 22 female) participated in the experiment, all with normal or corrected to normal vision, right handed and naive to the purpose of the study. The participants were randomly assigned to the tDCS (*n* = 15) and sham (*n* = 15) groups. They had no history of neuropsychiatric or visual diseases, no seizure episodes, no use of medications known to affect cortical excitability and no implants or lesions in the region of the head. The functioning mechanism and possible side effects of tDCS were described to participants, and all of them signed a written informed consent form according to the declaration of Helsinki guidelines, as approved by the institutional ethics committee of University of Haifa (307/15).

#### tDCS

Stimulation was delivered via a Neuro-Conn DC-Stimulator plus device. A current density of 0.04 mA/cm2 was used, 1 mA current dose for 15 min, through two sponge electrodes soaked in a saline solution (NaCl 0.9%). The active electrode (5 × 5 cm) was placed at left posterior occipital area: O-O1 (10–20 international system), while the return electrode (7 × 5 cm) was located at right orbitofrontal area Fp2. Non-conductive elastic bandage was used to keep the electrodes in place.

The stimulation started with current being gradually ramped from 0 to 1 mA in 30 s. For sham group, the current was ramped down to 0 precisely afterwards, while for tDCS group the current was ramped down from 1 to 0 mA in 30 s. at the end of stimulation (i.e., after 15 min). Mean electrical impedance and reported side effects during stimulation were logged for each participant.

#### Stimuli and procedure

The stimuli and procedure were similar to Experiment 1 except that anodal or sham stimulation was applied for 15 min during the practice phase, after which tDCS was removed and the test phase begun.

### Results

#### tDCS effects in the practice phase, during stimulation

**Figure 3:**
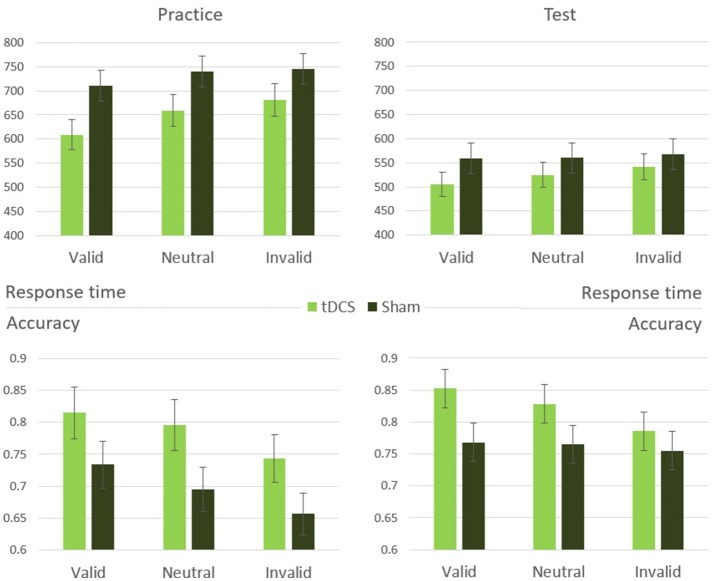
Experiment 2 results: response time and accuracy means and standard errors in the cueing conditions during practice and test stages.

A mixed-design two-way analysis of variance with stimulation (tDCS, sham) as a between-subjects variable and cueing condition (valid, neutral, invalid) as a within-subjects variable was performed on the response times of the practice phase. This analysis revealed a significant main effect of cueing [*F*_(2, 56)_ = 5.87, *p* = 0.008, η^2^ = 0.30]. The main effect of stimulation was marginally significant [*F*_(1, 28)_ = 3.36, *p* = 0.08, η^2^ = 0.11]. No interaction occurred between the stimulation and cueing manipulations [*F*_(2, 56)_ = 0.50, *p* = 0.61]. A similar analysis performed on accuracy yielded comparable results, with a significant main effect of cueing [*F*_(2, 56)_ = 10.33, *p* < 0.001, η^2^ = 0.43], a marginally significant difference between the stimulation types [*F*_(1, 28)_ = 3.72, *p* = 0.07, η^2^ = 0.12], and no significant interaction [*F*_(2, 56)_ = 0.25, *p* = 0.78; Figure 3].

#### tDCS effects in the test phase, after stimulation

A similar mixed-design two-way analysis of variance was performed on response time of trials belonging to the test phase. This analysis revealed a significant main effect of cueing [*F*_(2, 56)_ = 11.43, *p* < 0.001, η^2^ = 0.46]. Unlike the practice phase, here was no significant difference between stimulation types [*F*_(1, 28)_ = 0.79, *p* = 0.38], but a significant interaction emerged [*F*_(2, 56)_ = 3.46, *p* = 0.046, η^2^ = 0.20; Figure 3]. Performing the same analysis on the accuracy data revealed only a significant main effect of cueing [*F*_(2, 56)_ = 3.43, *p* = 0.047, η^2^ = 0.20], with no significant main effect of stimulation [*F*_(1, 28)_ = 1.74, *p* = 0.20] and no interaction [*F*_(2, 56)_ = 1.49, *p* = 0.24].

**Figure 4:**
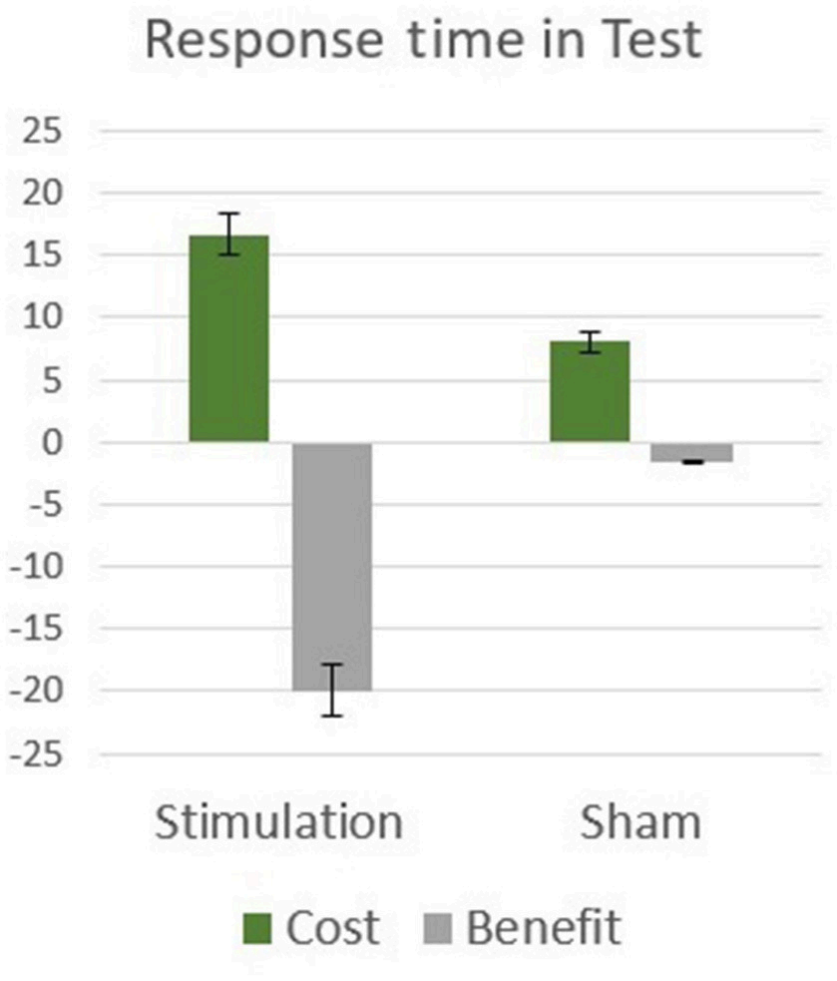
Experiment 2 results: attentional cost and benefit means and standard errors in response time during test.

To clarify the influence of tDCS stimulation in the test phase, independently of the attention manipulations, the tDCS and sham groups were compared in the neutral cue condition only, in which no attention bias was generated. An independent *t*-test comparison between the two groups indicated that they did not differ significantly [*t*_(28)_ = 0.80, *p* = 0.43], this indicates that the cueing-stimulation interaction found in the test phase is due to the differences in attention allocation between the groups. In order to further inspect the origins of the interaction between stimulation and spatial cueing, cost (neutral vs. invalid) and benefit (neutral vs. valid) measures were calculated and compared between the two stimulation groups. The influence of cueing was manifested considerably stronger in tDCS group as compared with the sham group. A mixed effects two-way analysis of variance (stimulation: tDCS, sham; attention: cost, benefit) performed on response time revealed a significant effect of cue [*F*_(1, 28)_ = 20.96, *p* < 0.001, η^2^ = 0.43], with no main effect for stimulation type [*F*_(1, 28)_ = 0.95, *p* = 0.34]. A significant interaction was found between stimulation and cue [*F*_(1, 28)_ = 7.11, *p* = 0.013, η^2^ = 0.20; Figure 4]. The results indicate that both cost and benefit effects were equally more pronounced in tDCS group compared to sham control group, highlighting the differences in attention allocation between the two groups.

## Discussion

In the present study we examined the influence of spatial attention on visual acuity, with and without tDCS. First, with regard to the general effect of spatial attention, we replicated the results of previous studies (e.g., Montagna et al., 2009): valid cues drew attention to the stimulus' location, enhancing its representation and improving performance, while invalid cues drew attention away, impairing visual acuity. The findings provide further support to the assertion that spatial attention can affect early visual processing of the attended target, including the resolution in which it is encoded. Second, regarding the overall effect of stimulation, anodal tDCS to posterior occipital area marginally enhanced overall performance during the stimulation course, as expressed by an improvement in response time and accuracy of the tDCS group compared to sham. This finding is consistent with previous studies demonstrating that tDCS of occipital regions affects visual processing. For instance, neuroenhancement of early visual areas was shown to improve visual hyperacuity (Reinhart et al., 2017), chromatic contrast sensitivity (Costa et al., 2012) and phosphene thresholds (Antal et al., 2003; Sczesny-Kaiser et al., 2016). The current findings are also highly concordant with the results of Olma et al. (2011) who used a comparable stimulation protocol to enhance activation in early visual areas, and attained sensitivity improvement in an orientation discrimination task. Our findings complement the claim by which anodal tDCS can increase visual sensitivity during stimulation course.

Most importantly, in the test phase (i.e., after the end of stimulation), we found a significant interaction between the cueing manipulation and stimulation types in response time. During this experimental phase both groups performed an identical task under identical conditions (i.e., both without stimulation), which enabled us to inspect the continuing effects of neuroenhancement. We found that tDCS magnified the effects of attention. Both effects of cost and benefit were larger in the tDCS group than in sham group. These finding are consistent with those of Mulckhuyse et al. (2011). In that study, TMS was applied to the occipital pole shortly before the onset of the target, which was either a horizontal or vertical line segment. The task was to indicate the target orientation. Prior to target onset, an abrupt onset cue marked one of the two possible target locations. In line with our findings, Mulckhuyse et al. found that a single-pulse TMS administered to the occipital pole contralateral to the stimulus magnified both effects of spatial attention—the benefit and the cost. Together, Mulckhuyse et al.'s study and ours provide compelling evidence in support of mutual influences of cortical stimulation and attention allocation on perception.

There are several, not mutually exclusive, ways in which the post-stimulation interaction can be explained. One possibility is that the spatial cues were perceived as more salient as a result of stimulation, and therefore were more efficient in drawing attention. This in turn, may have increased the efficiency of attention engagement at the cued locations, but also decreased the efficiency of attention disengagement from these locations (LaBerge, 1974). Another explanation regards the size of the attentional beam. Amplified by tDCS, the size of the attentional window might have become more precisely focused on the attended stimulus, compared with sham. A more narrow focus of attention could improve the encoding quality of information extracted from the attended location, but could also harm the encoding of information presented in another location.

The outcomes of the present experiment can also be interpreted in the context of the three attention systems proposed by Posner and Petersen (1990), which distinguish between alerting, orienting and executive attention operations. The three systems are signified by differential neurophysiological activations and performance (as revised by Posner and Rothbart, 2007). In this framework, warning and preparatory signals for incoming events are attention alerting, while orienting signals pre-specify the spatial location or source of the target event. Both alerting and orientating are stimuli driven, with mandatory and automatic processing and response (Posner and Rothbart, 2007; Kahneman, 2011). Executive control processes, on the other hand, represent top down operations, which involve the conduct of voluntary intentions and goals as well as monitoring and resolving inconsistencies and conflicts. In our experiment the + sign preceding the cue in each trial is alerting. Congruent cues are valid orienting and incongruent cues call for executive control involvement to redirect attention. Accordingly, neutral cues served as an alert only and did not benefit or misdirect location orientation. The results presented in Figure 4 show that when compared to neutral trial response times, the tDCS group had significantly more pronounced benefit for congruent cues and larger cost for incongruent cues calling for executive control. Thus, the tDCS group shows differential effects of both automatic and controlled categories of attention. Namely, automatic attention capturing by cues (orientation) and effortful redirection of attention to overcome incongruence (executive control).

Previous studies suggest that the mutual influences of attention and neurostimulation can also be interpreted as attentional modulation of the stimulation outcomes. Specifically, TMS studies have shown that stimulation of the visual cortex in different attention states leads to dissimilar perceptual results. For instance, Bestmann et al. (2007) produced illusory percepts of light (phosphenes) by applying TMS over the visual cortex. They found that at the attended locations a lower TMS intensity was needed to create a phosphene, compared to unattended locations. As another example, Ruff et al. (2007) showed that TMS stimulation of the right intraparietal sulcus (implicated in attention) elicited a pattern of activity changes in the visual cortex that strongly depended on current visual context. Such effects are often referred to as state dependent effects of stimulation and were recently reviewed by Romei et al. (2016). According to the state-dependence principal, the neural representation of a stimulus is determined by its properties, neurostimulation characteristics, and by the initial state of the stimulated brain area (Silvanto et al., 2008a,b). According to this principal, the joint effects observed in our study reflect the interaction between initial brain excitability and the external input—attention cueing and stimulation. Namely, the increased excitability of the early visual area, brough about by spatial attention, may have enhanced the impact of brain stimulation (e.g., Silvanto and Cattaneo, 2017).

As mentioned above, even though the task and experimental conditions in the post-stimulation test phase were identical for both tDCS and sham groups, attention performance varied significantly between the two groups. Post-stimulation effects found in Experiment 2 are consistent with tDCS literature. For example, Falcone et al. (2012) showed that tDCS enhances visual sensitivity over a period of 24 h following the stimulation. In the auditory domain, Garin et al. (2011) revealed a significant effect of anodal tDCS on tinnitus suppression. In their study half the patients declared long-term effects, some lasting more than 2 weeks after the tDCS session. Lastly, Au et al. (2016) reported that tDCS enhances visuospatial memory, and that the achieved results are preserved for several months after experiment completion. Since tDCS does not induce action potentials, but seems to merely influence the probability to spontaneously create them (Stagg and Nitsche, 2011), enhancing activity in the area of interest by performing a task during stimulation is thought to be an effective way to induce substantial and lasting effects (Costa et al., 2015). Accordingly, it was found that tDCS influences not only task learning but also its consolidation (e.g., Peters et al., 2013; Richmond et al., 2014). Liebetanz et al. (2002) and Nitsche et al. (2003, 2004) showed that tDCS after-effects can be expressed in a reduced concentration of GABA neurotransmitter and receptors, and in formation and functioning of NMDA receptors. Moreover, Stagg et al. (2009) and Clark et al. (2011) showed changes in glutamate concentrations after tDCS - deducing that tDCS may function as an accelerator of a Long Term Potentiation or Depression like mechanisms in the stimulated area.

Neural alterations of the stimulated area are not the only explanation of the longer-lasting effects achieved in Study 2. According to Nitsche et al. (2008) tDCS effects appear to be site specific but not site limited. Respectively, a vast field of studies connects between functionality of early visual areas, and areas which mediate attention allocation (e.g., Greenberg et al., 2012). There is a fair possibility that the achieved interaction depicts a change in functional connectivity between early visual and attention areas. Such a change in connectivity may involve not only forward but also feedback connections to early visual areas. According to predictive coding models (revised by Van der Helm, 2016), human brain corrects error in a cascade of processing. Namely, higher-level cortical systems attempt to predict the inputs to lower-level systems. Errors in prediction cause higher-level systems to adapt so as to reduce the discrepancy. This model of processing was shown to be reliable regarding the functioning of early visual areas (e.g., Murray et al., 2002; Fang et al., 2008; Alink et al., 2010). One could speculate that in the current study, the deviance between the predicted neural input and the obtained input necessary for creating neural firing in early visual areas, was lower in tDCS group due to membrane depolarization. This way spontaneous firing was initiated more easily and the functional connectivity between early visual areas and the preceding areas changed. Systematic training of this enhanced functioning might have caused a continuous change in neural connectivity, differentiating between divergent cueing conditions, and resulting in a consistent response in post-stimulation trials.

### Limitations and future research

Though quite informative in cognitive findings, the current study emphasizes the gap in understanding of tDCS influence on neural functioning of visual attention. A few theories were suggested, but in order to gain a comprehensive understanding one must bridge the gap between well-established cognitive background and the recent neurobiological findings. During the last decades, theories from cognitive and perceptual fields received additional support by means of new technological approaches. Some of these techniques provide decent precision both temporally and spatially (e.g., Crone et al., 1998). Using suitable technology can help to test the hypotheses regarding the exact neural functioning behind the observed cognitive changes. To improve the understanding of tDCS influence on the processes of learning, longitudinal studies might be performed. Stimulating the areas of interest at different time-points of learning can help answering a variety of fascinating questions regarding gradual improvement of stimuli representation.

As the current study examined the influence of anodal tDCS on visual attention, the adverse influence of cathodal tDCS might be considered in future studies. Cathodal tDCS was previously shown to impair performance in visual tasks, functioning via neuroinhibition (reviewed by Costa et al., 2015). Results of the current study can be further advanced by examining the interaction between cathodal stimulation and spatial cueing. Such a manipulation may yield cross-validated results to the hypothesized mechanisms. Will the effects of spatial cues remain or will they disappear? Will cathodal stimulation influence the overall sensitivity, diminishing visual acuity? While it can be speculated that inhibition tDCS mechanisms function as an inversed version of the excitation processes, the neural evidence of earlier studies suggests otherwise (Matsunaga et al., 2004). Understanding the reducing effects of cathodal stimulation can shed more light on functional connectivity and interdependence between networks which mediate visual attention. Posner and Rothbart (2007) developed the attention network test (ANT), which aims to explore the underlying neural networks of alerting, orienting, and executive control. Using this paradigm, combined with tDCS stimulation to early visual area can clarify the origin of the achieved effects, presumably by highlighting the independent components of orienting and executive control.

## Conclusion

Three clear findings emerged from the present study. First, anodal tDCS improved spatial acuity measured with a Landolt gap task, likely via stimulus enhancement. Second, allocating spatial attention to the stimulus location also improved performance, while allocation of attention to the wrong location impaired performance. Third, attention allocation interacted with tDCS, becoming more prominent after stimulation. The effects of spatial attention as well as the initial gain in performance are consistent with previous attention-related and tDCS literature and could be explained by an improvement of effective cells sensitivity thresholds. The latter findings imply mutual interaction between attentional and tDCS effects. These effects might result from alterations in the stimulated area, or a change in relations between early visual and functionally connected attention areas.

## Author contributions

TB: designed the study, acquired, analyzed and interpreted the data, and drafted the article; DG and YY: designed the study, interpreted data, and revised the manuscript.

### Conflict of interest statement

The authors declare that the research was conducted in the absence of any commercial or financial relationships that could be construed as a potential conflict of interest.

## References

[B1] Alink A. Schwiedrzik C. M. Kohler A. Singer W. Muckli L. (2010). Stimulus predictability reduces responses in primary visual cortex. J. Neurosci. 30, 2960–2966. 10.1523/JNEUROSCI.3730-10.2010 20181593 PMC6633950

[B2] Antal A. Kincses T. Z. Nitsche M. A. Paulus W. (2003). Modulation of moving phosphene thresholds by transcranial direct current stimulation of V1 in human. Neuropsychologia 41, 1802–1807. 10.1016/S0028-3932(03)00181-7 14527543

[B3] Antal A. Nitsche M. A. Paulus W. (2001). External modulation of visual perception in humans. Neuroreport 12, 3553–3555. 10.1097/00001756-200111160-00036 11733710

[B4] Au J. Katz B. Buschkuehl M. Bunarjo K. Senger T. Zabel C.. (2016). Enhancing working memory training with transcranial direct current stimulation. J. Cogn. Neurosci. 28, 1419–1432. 10.1162/jocn_a_00979 27167403

[B5] Bestmann S. Ruff C. C. Blakemore C. Driver J. Thilo K. V. (2007). Spatial attention changes excitability of human visual cortex to direct stimulation. Curr. Biol. 17, 134–139. 10.1016/j.cub.2006.11.063 17240338 PMC1815217

[B6] Clark V. P. Coffman B. A. Trumbo M. C. Gasparovic C. (2011). Transcranial direct current stimulation (tDCS) produces localized and specific alterations in neurochemistry: a 1 H magnetic resonance spectroscopy study. Neurosci. Lett. 500, 67–71. 10.1016/j.neulet.2011.05.244 21683766

[B7] Costa T. L. Lapenta O. M. Boggio P. S. Ventura D. F. (2015). Transcranial direct current stimulation as a tool in the study of sensory-perceptual processing. Atten. Percept. Psychophys. 70, 1813–1840. 10.3758/s13414-015-0932-3 26139152

[B8] Costa T. L. Nagy B. V. Barboni M. T. Boggio P. S. Ventura D. F. (2012). Transcranial direct current stimulation modulates human color discrimination in a pathway-specific manner. Front. Psychiatry 3:78. 10.3389/fpsyt.2012.00078 22988446 PMC3439847

[B9] Creutzfeldt O. D. Fromm G. H. Kapp H. (1962). Influence of transcortical de-currents on cortical neuronal activity. Exp. Neurol. 5, 436–452. 10.1016/0014-4886(62)90056-0 13882165

[B10] Crone N. E. Miglioretti D. L. Gordon B. Lesser R. P. (1998). Functional mapping of human sensorimotor cortex with electrocorticographic spectral analysis. II. Event-related synchronization in the gamma band. Brain 121, 2301–2315. 10.1093/brain/121.12.2301 9874481

[B11] Falcone B. Coffman B. A. Clark V. P. Parasuraman R. (2012). Transcranial direct current stimulation augments perceptual sensitivity and 24-hour retention in a complex threat detection task. PLoS ONE 7:e34993. 10.1371/journal.pone.0034993 22511978 PMC3325218

[B12] Fang F. Boyaci H. Kersten D. Murray S. O. (2008). Attention-dependent representation of a size illusion in human V1. Curr. Biol. 18, 1707–1712. 10.1016/j.cub.2008.09.025 18993076 PMC2638992

[B13] Fiorentini A. Berardi N. (1981). Learning in grating waveform discrimination: specificity for orientation and spatial frequency. Vision Res. 21, 1149–1158. 10.1016/0042-6989(81)90017-1 7314493

[B14] Garin P. Gilain C. Van Damme J. P. de Fays K. Jamart J. Ossemann M.. (2011). Short-and long-lasting tinnitus relief induced by transcranial direct current stimulation. J. Neurol. 258, 1940–1948. 10.1007/s00415-011-6037-6 21509429 PMC3214608

[B15] Greenberg A. S. Verstynen T. Chiu Y. C. Yantis S. Schneider W. Behrmann M. (2012). Visuotopic cortical connectivity underlying attention revealed with white-matter tractography. J. Neurosci. 32, 2773–2782. 10.1523/JNEUROSCI.5419-11.2012 22357860 PMC3321828

[B16] Guo K. Robertson R. G. Pulgarin M. Nevado A. Panzeri S. Thiele A.. (2007). Spatio-temporal prediction and inference by V1 neurons. Eur. J. Neurosci. 26, 1045–1054. 10.1111/j.1460-9568.2007.05712.x 17714195

[B17] Kahneman D. (2011). Thinking, Fast and Slow. New York, NY: Farrar, Straus and Giroux.

[B18] Kastner S. Pinsk M. A. De Weerd P. Desimone R. Ungerleider L. G. (1999). Increased activity in human visual cortex during directed attention in the absence of visual stimulation. Neuron 22, 751–761 10.1016/S0896-6273(00)80734-5 10230795

[B19] LaBerge D. (1974). Identification of two components of the time to switch attention: a test of a serial and a parallel model of attention, in Attention and Performance ZV, ed Kornblum S. (New York, NY: Academic Press), 71–85.

[B20] Liebetanz D. Nitsche M. A. Tergau F. Paulus W. (2002). Pharmacological approach to the mechanisms of transcranial DC-stimulation-induced after-effects of human motor cortex excitability. Brain 125, 2238–2247. 10.1093/brain/awf238 12244081

[B21] Nitsche M. A. Fricke K. Henschke U. Schlitterlau A. Liebetanz D. Lang N.. (2003). Pharmacological modulation of cortical excitability shifts induced by transcranial direct current stimulation in humans. J. Physiol. 553, 293–301. 10.1113/jphysiol.2003.049916 12949224 PMC2343495

[B22] Nitsche M. A. Liebetanz D. Schlitterlau A. Henschke U. Fricke K. Frommann K.. (2004). GABAergic modulation of DC stimulation-induced motor cortex excitability shifts in humans. Eur. J. Neurosci. 19, 2720–2726. 10.1111/j.0953-816X.2004.03398.x 15147306

[B23] Matsunaga K. Nitsche M. A. Tsuji S. Rothwell J. C. (2004). Effect of transcranial DC sensorimotor cortex stimulation on somatosensory evoked potentials in humans. Clin. Neurophysiol. 115, 456–460. 10.1016/S1388-2457(03)00362-6 14744588

[B24] Mayfrank L. Kimmig H. Fischer B. (1987). The role of attention in the preparation of visually guided saccadic eye movements in man, in Eye Movements from Physiology to Cognition, eds O'Regan J. K. Levy-Schoen A. (New York, NY: Elsevier), 37–45.

[B25] Montagna B. Pestilli F. Carrasco M. (2009). Attention trades off spatial acuity. Vision Res. 49, 735–745. 10.1016/j.visres.2009.02.001 19385088 PMC3375052

[B26] Mulckhuyse M. Kelley T. A. Theeuwes J. Walsh V. Lavie N. (2011). Enhanced visual perception with occipital transcranial magnetic stimulation. Eur. J. Neurosci. 34, 1320–1325. 10.1111/j.1460-9568.2011.07814.x 21848918 PMC3410532

[B27] Murray S. O. Kersten D. Olshausen B. A. Schrater P. Woods D. L. (2002). Shape perception reduces activity in human primary visual cortex. *Proc. Natl. Acad. Sci*. U.S.A. 99, 15164–15169. 10.1073/pnas.192579399 PMC137561 12417754

[B28] Nitsche M. A. Cohen L. G. Wassermann E. M. Priori A. Lang N. Antal A.. (2008). Transcranial direct current stimulation: state of the art 2008. Brain Stimul. 1, 206–223. 10.1016/j.brs.2008.06.004 20633386

[B29] Olma M. C. Kraft A. Roehmel J. Irlbacher K. Brandt S. A. (2011). Excitability changes in the visual cortex quantified with signal detection analysis. Restor. Neurol. Neurosci. 29, 453–461. 10.3233/RNN-2011-0607 22278016

[B30] Peters M. A. Thompson B. Merabet L. B. Wu A. D. Shams L. (2013). Anodal tDCS to V1 blocks visual perceptual learning consolidation. Neuropsychologia 51, 1234–1239. 10.1016/j.neuropsychologia.2013.03.013 23562964

[B31] Posner M. I. Petersen S. E. (1990). The attention system of the human brain. Annu. Rev. Neurosci. 13, 25–42. 10.1146/annurev.ne.13.030190.000325 2183676

[B32] Posner M. I. Rothbart M. K. (2007). Research on attention networks as a model for the integration of psychological science. Annu. Rev. Psychol. 58, 1–23. 10.1146/annurev.psych.58.110405.085516 17029565

[B33] Reinhart R. M. Cosman J. D. Fukuda K. Woodman G. F. (2017). Using transcranial direct-current stimulation (tDCS) to understand cognitive processing. Atten. Percept. Psychophys. 79, 3–23. 10.3758/s13414-016-1224-2 27804033 PMC5539401

[B34] Richmond L. L. Wolk D. Chein J. Olson I. R. (2014). Transcranial direct current stimulation enhances verbal working memory training performance over time and near transfer outcomes. J. Cogn. Neurosci. 26, 2443–2454. 10.1162/jocn_a_00657 24742190

[B35] Romei V. Thut G. Silvanto J. (2016). Information-based approaches of noninvasive transcranial brain stimulation. Trends Neurosci. 39, 782–795. 10.1016/j.tins.2016.09.001 27697295

[B36] Ruff C. C. Bestmann S. Blankenburg F. Bjoertomt O. Josephs O. Weiskopf N.. (2007). Distinct causal influences of parietal versus frontal areas on human visual cortex: evidence from concurrent TMS–fMRI. Cereb. Cortex 18, 817–827. 10.1093/cercor/bhm128 17652468 PMC2601025

[B37] Sczesny-Kaiser M. Beckhaus K. Dinse H. R. Schwenkreis P. Tegenthoff M. Höffken O. (2016). Repetitive transcranial direct current stimulation induced excitability changes of primary visual cortex and visual learning effects—a pilot study. Front. Behav. Neurosci. 10:116. 10.3389/fnbeh.2016.00116 27375452 PMC4891342

[B38] Silvanto J. Cattaneo Z. (2017). Common framework for “virtual lesion” and state-dependent TMS: the facilitatory/suppressive range model of online TMS effects on behavior. Brain Cogn. 119, 32–38. 10.1016/j.bandc.2017.09.007 28963993 PMC5652969

[B39] Silvanto J. Cattaneo Z. Battelli L. Pascual-Leone A. (2008a). Baseline cortical excitability determines whether TMS disrupts or facilitates behavior. J. Neurophysiol. 99, 2725–2730. 10.1152/jn.01392.2007 18337360 PMC3533239

[B40] Silvanto J. Muggleton N. Walsh V. (2008b). State-dependency in brain stimulation studies of perception and cognition. Trends Cogn. Sci. 12, 447–454. 10.1016/j.tics.2008.09.004 18951833

[B41] Stagg C. J. Best J. G. Stephenson M. C. O'Shea J. Wylezinska M. Kincses Z. T.. (2009). Polarity-sensitive modulation of cortical neurotransmitters by transcranial stimulation. J. Neurosci. 29, 5202–5206. 10.1523/JNEUROSCI.4432-08.2009 19386916 PMC6665468

[B42] Stagg C. J. Nitsche M. A. (2011). Physiological basis of transcranial direct current stimulation. Neuroscientist 17, 37–53. 10.1177/1073858410386614 21343407

[B43] Van der Helm P. A. (2016). Structural coding versus free-energy predictive coding. Psycho. Bull. Rev. 23, 663–677. 10.3758/s13423-015-0938-9 26407895

[B44] Westheimer G. (2001). Is peripheral visual acuity susceptible to perceptual learning in the adult?. Vision Res. 41, 47–52. 10.1016/S0042-6989(00)00245-5 11163615

[B45] Yeshurun Y. Carrasco M. (1998). Attention improves or impairs visual performance by enhancing spatial resolution. Nature 396, 72–75. 10.1038/23936 9817201 PMC3825508

[B46] Yeshurun Y. Carrasco M. (1999). Spatial attention improves performance in spatial resolution tasks. Vision Res. 39, 293–306. 10.1016/S0042-6989(98)00114-X 10326137

[B47] Yeshurun Y. Rashal E. (2010). Precueing attention to the target location diminishes crowding and reduces the critical distance. J. Vision 10:16. 10.1167/10.10.16 20884481

